# Using SBAR in psychiatry: findings from two london hospitals

**DOI:** 10.1192/bjo.2021.530

**Published:** 2021-06-18

**Authors:** Benjamin Janaway, Ruslan Zinchenko, Lubna Anwar, Clare Wadlow, Edwin Soda, Karen Cove

**Affiliations:** 1 Barnet, Enfield and Haringey Mental Health Trust; 2 Enfield South Locality Team (Lucas House)

## Abstract

**Aims:**

We aimed to evaluate the use of the Situation, Background, Assessment and Recommendation communication tool (SBAR) at two large psychiatric hospitals, in order to design new approaches to teach and reinforce its sustained use. In doing so we hope to improve communication, staff experience and outcomes for patients.

We hypothesised that use prior to intervention would be low and attitudes inconsistent between teams and objective data.

**Background:**

SBAR is a communication tool developed to accurately refer information with improved outcomes within the NHS. Within psychiatry there is evidence of relatively poor care of medical problems leading to adverse outcomes in a group more susceptible to multiple physical illnesses. The reasons for this include a cultural ethos of learned helplessness in staff and lack of medical knowledge.

The use of SBAR is likely to overcome these issues.

**Method:**

Surveys were presented to doctors and nurses staff at two Psychiatric Hospitals, Chase Farm and Edgeware. Inclusion in the survey was voluntary and anonymous. Questions elucidated topics ranging from awareness of SBAR through to its use and benefits.

Objective data were also collected, looking at handover gathered during the survey period. This was collected via phone from the duty physician over a five-day period, twice-daily. Qualitative data on handover content was collected at CFH.

Audit standards around knowledge, use and outcomes were set. Data were collected and analysed in house.

**Result:**

The data (n23) showed that most nurses reported awareness (86.96%) ease of use (86.96%) actual use (60.87%) efficacy in communication (78.26%) value in understanding patients (78.26%) and agreement with mandatory use (78.26%.)

Doctor reports (n14) showed that although 100% were aware of SBAR, no respondents thought nurse-led communication was adequate, or that SBAR was used. The majority thought that mandatory SBAR use would improve communication (92.86%) and patient care (100%)

Objective data (pooled) of referrals showed that on 6.52% used SBAR. Qualitative data showed that handover was often inaccurate, lacking in information and unsafe. Suggestions for teaching included written or video media, or taught classes.

All audit standards were failed.

**Conclusion:**

SBAR is an effective tool for improving communication and patient outcomes, and is well perceived by the MDT. However, it is poorly used with psychiatry leading to adverse outcomes. Reported use is undermined by objective data. Its mandatory use is well supported and new teaching initiatives are thus being designed to remedy this and improve client experience.

